# Accuracy of digital impressions versus conventional impressions for 2 implants: an in vitro study evaluating the effect of implant angulation

**DOI:** 10.1186/s40729-021-00355-6

**Published:** 2021-07-30

**Authors:** Jaafar Abduo, Joseph E. A. Palamara

**Affiliations:** 1 Prosthodontics Department, Clinical Dentistry (Implants), Melbourne Dental School, Melbourne University, 720 Swanston Street, Melbourne, Victoria 3010 Australia; 2 Restorative Section, Melbourne Dental School, Melbourne University, 720 Swanston Street, Melbourne, Victoria 3010 Australia

**Keywords:** Implant impression, Precision, Scanning, Splinted, Trueness

## Abstract

**Background:**

Accurate implant impression is an essential requirement for the fabrication of implant prosthesis. This in vitro study evaluated the accuracy of digital impressions by intraoral scanner (IOS) systems in comparison to conventional impressions for recording the position of 2 parallel implants and 2 divergent implants.

**Materials and methods:**

In vitro 3-unit prosthesis master models with 2 tissue level implants were fabricated; one model had parallel implants, and the other model had one 15° tilted implant. The conventional open-tray impressions were obtained with non-splinted (NSP) and splinted (SP) impression copings. Trios 4 (TS), Medit i500 (MT), and True Definition (TD) were used to make digital impressions with scan bodies. A total of 10 impressions were obtained with every technique. The virtual test images of the conventional and digital impressions were converted to 2 virtual implant images. For each group, trueness, precision, inter-implant distance deviation, and angle deviation were measured.

**Results:**

There was a general tendency for digital impressions to provide a more accurate outcome for trueness, precision, and angle deviation. The 2 conventional impressions showed similar accuracy, except for the angle deviation, where the NSP was significantly inferior than SP (*p* < 0.01) for the divergent implants model. The TD was generally the least accurate among all the IOS systems, especially for the inter-implant distance deviation (*p* < 0.05).

**Conclusions:**

Within the limitations of the laboratory set-up of the present study and the limited clinical resemblance, the digital impressions appeared to have sufficient accuracy for 2 implants and were least affected by the presence of angle between implants. The most inferior outcome was observed for the NSP technique.

## Background

Accurate implant impression is an essential step prior to implant prosthesis fabrication. As the integrated implants are rigidly anchored in alveolar bone, inaccuracies in the implant impression will compromise the fit of implant prosthesis and may result in biological and mechanical complications [1, 2]. The conventional implant impression procedure involves recording the implant position using an impression coping, elastomeric material, and a rigid tray. The literature disclosed several factors that influence the accuracy of conventional impression such as implants number, angulation, depth, impression technique, and impression material [3–5]. To enhance the accuracy of implant impression, several technique modifications were proposed. This involved splinting impression copings, modifying impression copings, tray design, and use of more rigid material [3, 4, 6–8]. While the additional steps in implant impression, such as splinting impression copings, showed tendency to improve implant impression accuracy [3, 4], they are technique sensitive and involve additional materials and more clinical time.

Recently, with the advancement of digital technologies, digital impression became a feasible alternative to record implants position. With this approach, a scan body is attached on the implant, and an intraoral scanner (IOS) is used to record the scan body position in addition to the adjacent teeth and the surrounding tissue [9–11]. Subsequently, a virtual image of the scan body and the surrounding structure is generated. The scan body surface of the virtual image is used to determine the implant position with the aid of a digital library compatible with the scan body and the implant brand [12]. This is followed by either virtual design and fabrication of the implant prosthesis or production of dental cast on which the prosthesis is fabricated [12–14]. There has been several studies on the use of IOS systems for implant digital impressions [15–20]; however, since the IOS systems are continuously changing, frequent research to validate their accuracy is necessary [21–24]. In addition, the accuracy of digital implant impression can be influenced by several factors such as implant angulation and depth, span length, location in the mouth, and number of implants. Therefore, the aim of this study was to evaluate the accuracy of digital impressions by multiple IOS systems in recording the position of 2 implants in comparison to conventional impression techniques. In addition, the study evaluated the effect of presence of clinically relevant degrees of divergence between the 2 implants. To simulate a routine IOS workflow, the experiment implemented reverse engineering of the recorded scan bodies for the purpose of direct evaluation of the virtual implants position. This is different from the majority of the published studies that evaluated the accuracy of the scanned surface of the scan bodies with or without the surrounding tissue [15, 21–23, 25]. The relevance of this step is that the virtual implants position is the determining factor of the accuracy of any impression technique. The null hypotheses where there is no difference between the different digital impressions and the conventional implant impressions, and there is no effect of the presence of divergence angle between the 2 implants.

## Methods

**Fig. 1:**
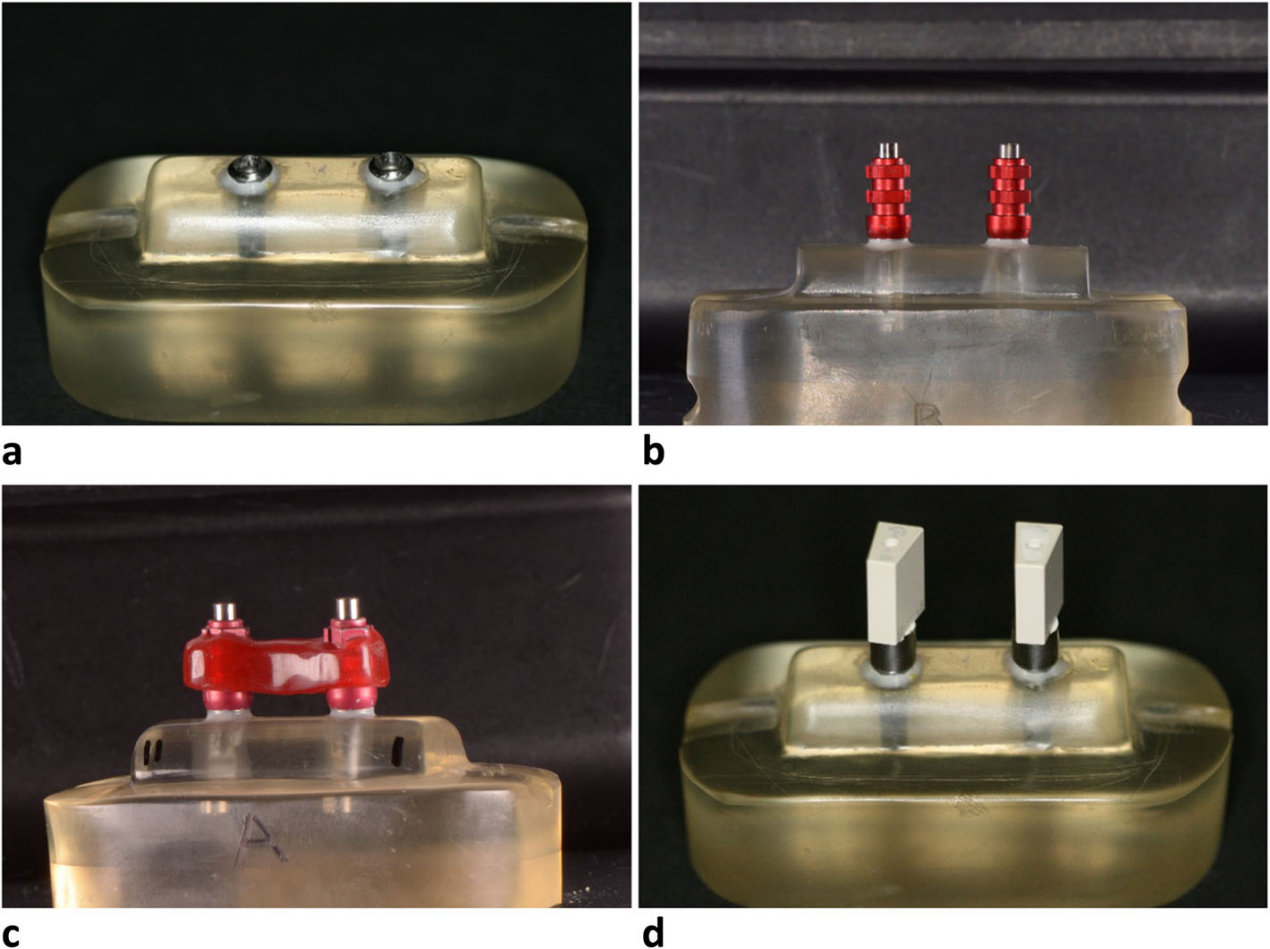
One of the master models. **a** A master model with a simulated healed ridge and 2 parallel implants. **b** The master model with separate impression copings for the NSP impression technique. **c** The impression copings were splinted for the SP impression technique. **d** The model with the scan bodies attached to the implants prior to scanning

Two master models of 2 implants placed to support 3-unit prostheses were fabricated. The models resembled a healed ridge of 8 mm width (Fig. 1a). Straumann tissue level regular implants of 4.8 mm neck diameter (Institut Straumann AG, Basel, Switzerland) were used and the models’ material was self-curing polymethylmethacrylate resin (Vertex Selfcuring Resin; Henry Schein, Waterloo, NSW, Australia). For the first model, the 2 implants were parallel. The other model had one divergent implant, where the implant was tilted by 15° angle buccolingually. The 2 models had similar dimensions, and the distance between the centers of the 2 implants was 15 mm. The implant platforms were located 1 mm above the simulated ridge, and the undercut regions beyond the implant platform was sealed with resin material. This ensured the tissue level implants platform was located at the crest of the ridge and to prevent impression material flow in the undercuts under the implant platform.

Two conventional impression groups were included in the study, non-splinted (NSP) and splinted (SP). These conventional techniques were chosen because they are commonly used for 3-unit prosthesis impression [3, 4]. Custom trays were fabricated for the 2 groups from light-cured acrylic resin material (Vertex Dental, Soesterberg, Netherlands). On the master models, 2 baseplate wax layers were applied to ensure a uniform space for impression material. Handles were included in the tray design at the mesial and distal aspects of the trays. The trays were indexed against the base of the models to control the seating of the trays during the impression procedure. For the NSP impression, the trays were designed with 2 round openings at the location of the implants. The diameters of the opening were approximately 2 mm wider than the impression copings. For the SP impressions, the 2 rounded openings were merged to provide space for the splinted impression copings. The impression copings for the SP technique were connected by self-curing acrylic resin material (GC Pattern Resin, GC Corp, Tokyo, Japan). The resin splints were at least 3 mm thick with an approximate thickness of 2 mm around the impression copings. Following resin polymerization, the splints were sectioned and re-joined by a freshly mixed resin [4]. The internal surface of the trays, the impression copings and the splints were painted by tray adhesive (VPS Tray adhesive, Kerr Corporation, Orange, CA, USA), and the trays openings were sealed by baseplate wax layer. All the conventional impressions were made by heavy body polyvinylsiloxane impression material (Kerr Extrude Extra type 1, Kerr Corporation). The impressions were removed after at least 10 min to ensure the material is completely set according to manufacturer recommendations. Implant replicas were connected to the impression copings, and the impressions were poured up using type IV dental stone (GC Fujirock EP, GC Corp., Tokyo, Japan). After 24 h, the casts were separated from the impressions. The impression making and pouring procedures were performed at room temperature. A total of 10 impressions were made for every model by each impression technique. The sample number was confirmed by power calculation through the G*Power software (version 3.1.9.2; University of Dusseldorf, Dusseldorf, Germany). By using the estimated accuracy variation between the different impression techniques [5, 8, 14], and applying 80% statistical power and 5% significance level, at least 8 impressions were needed for every technique.

For the digital impressions, scan bodies (ZFX Scan body, ZFX Dental, Zimmer Biomet, Warsaw, IN, USA) that are compatible with Straumann tissue level implants were attached on the implants. Three intraoral scanners were used to scan the master models: Trios 4 (TS) (3Shape, Copenhagen, Denmark), Medit i500 (MT) (MEDIT Corp, Seoul, Korea), and True Definition (TD) (3M ESPE, Seefeld, Germany) (Table 1). The manufacturers’ instructions were followed for all the scanners. This involved scanning the 2 scanning bodies in a zigzag motion to record the occlusal aspects followed by the lateral surfaces. The scanning commenced with TS and MT as they do not involve powder application. TD scanning required light powder application on the scan bodies. For every digital impression, a total of 10 scans were obtained for each master model. All the scans were exported into STL format, and were subsequently used as virtual test images.

**Table 1:** Details of the used IOS Systems for Digital Impressions

IOS system	Manufacturer details	Scanning mode
TS	3Shape, Copenhagen, Denmark	Ultrafast imaging based on confocal microscopy principles
MT	MEDIT Corp, Seoul, Korea	3D in-motion video recording technology
TD	3M ESPE, Seefeld, Germany	3D in-motion video recording technology

**Fig. 2:**
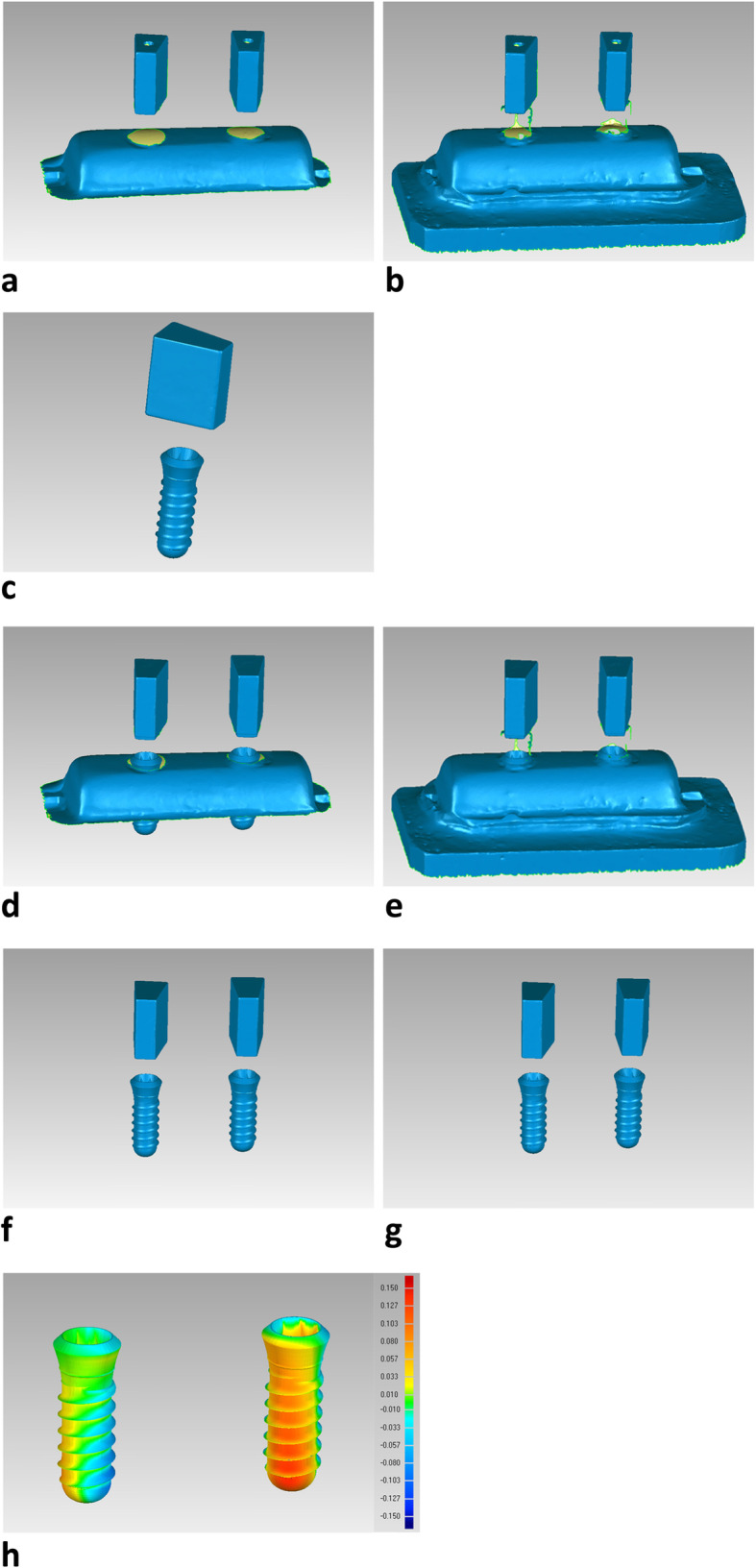
An example of the reverse engineering of the virtual reference master model image and virtual test model image before accuracy evaluation. **a** Virtual reference master model image. **b** Virtual test model image. **c** A virtual scan body with the parametric implant design was used for reverse engineering. **d** Virtual scan bodies with the virtual implants were superimposed against the virtual reference master model image. **e** Virtual scan bodies with the virtual implants were superimposed against the virtual test model image. **f** The reconstructed master model image with the implants after removal of the irrelevant surfaces. **g** The reconstructed test model image with the implants after removal of the irrelevant surfaces. **h** The remaining virtual implants were superimposed to measure the deviations between the 2 models

The master models with attached scan bodies were scanned by a laboratory scanner (Identica T300, Medit Identica, DT Technologies, Davenport, IA, USA) (Fig. 1b) to generate virtual reference images on which subsequent comparisons were conducted. Scan bodies were attached in the implant replicas of the stone casts of the conventional impressions, and were scanned by the laboratory scanner. This generated virtual test images of the conventional impression casts. A virtual scan body with a virtual implant was used to reverse engineer all the virtual reference test images. Subsequently, each image was converted to 2 virtual implants only (Fig. 2) without the surrounding structure.

Four accuracy variables were measured for every group: trueness, precision, inter-implant distance deviation, and angle deviation. All the measurements were conducted via a 3D analysis software (Geomagic Studio, 3D systems, Rock Hill, SC, USA). The trueness refers to the deviation of the implants of the test images from the reference image, and it quantifies the errors introduced from each impression technique. It involved superimposing the implants of each test image on the implants of the reference image. This was done by point to point registration followed by global registration that measured deviation between the different surfaces of the implants in micrometer. The absolute deviation of approximately 2000 random points on the implants surfaces was used to calculate the root mean square (RMS) value using the following equation:

$$ RMS=\sqrt{{\frac{\sum \left({R}_i-{C}_i\right)}{n}}^2} $$

where *R*_*i*_ is the spatial point of the reference image, *C*_*i*_ is the same spatial point of the test image, and *n* is the total number of points.

The less the magnitude, the greater the trueness and similarity to the master model. Precision is the deviation between the implants of the different test images within the same group, where it provides an indication of the repeatability of each technique. Therefore, for every group, a total of 45 precision measurements were obtained. The RMS values were measured similar to the trueness. The less the magnitude, the greater the precision and reproducibility of each technique.

The inter-implant distance was measured virtually between the centers of the implant platforms of each virtual image. The inter-implant distance deviation was the difference between the inter-implant distances of the reference and test images. The angle deviation was measured following superimposition of the implants against the implants of the reference image. For each implant, the maximum angle deviation was measured in degrees (°).

The mean and standard deviation of the variables of every impression technique were calculated for each model. The Shapiro-Wilks test was used to confirm normality of the date. The one-way ANOVA test followed by Tukey HSD post hoc test was applied for each master model to evaluate the difference among the different impression groups. For each impression group, the divergent implant models were compared against the parallel implant models using the *t* test. In addition, a two-way ANOVA test was implemented to evaluate the interaction between the impression technique and implants divergence for every evaluated variable. All the tests were performed using a statistics program (SPSS for Windows, v23; SPSS Inc., Chicago, IL, USA), with a 0.05 level of significance.

## Results

**Fig. 3:**
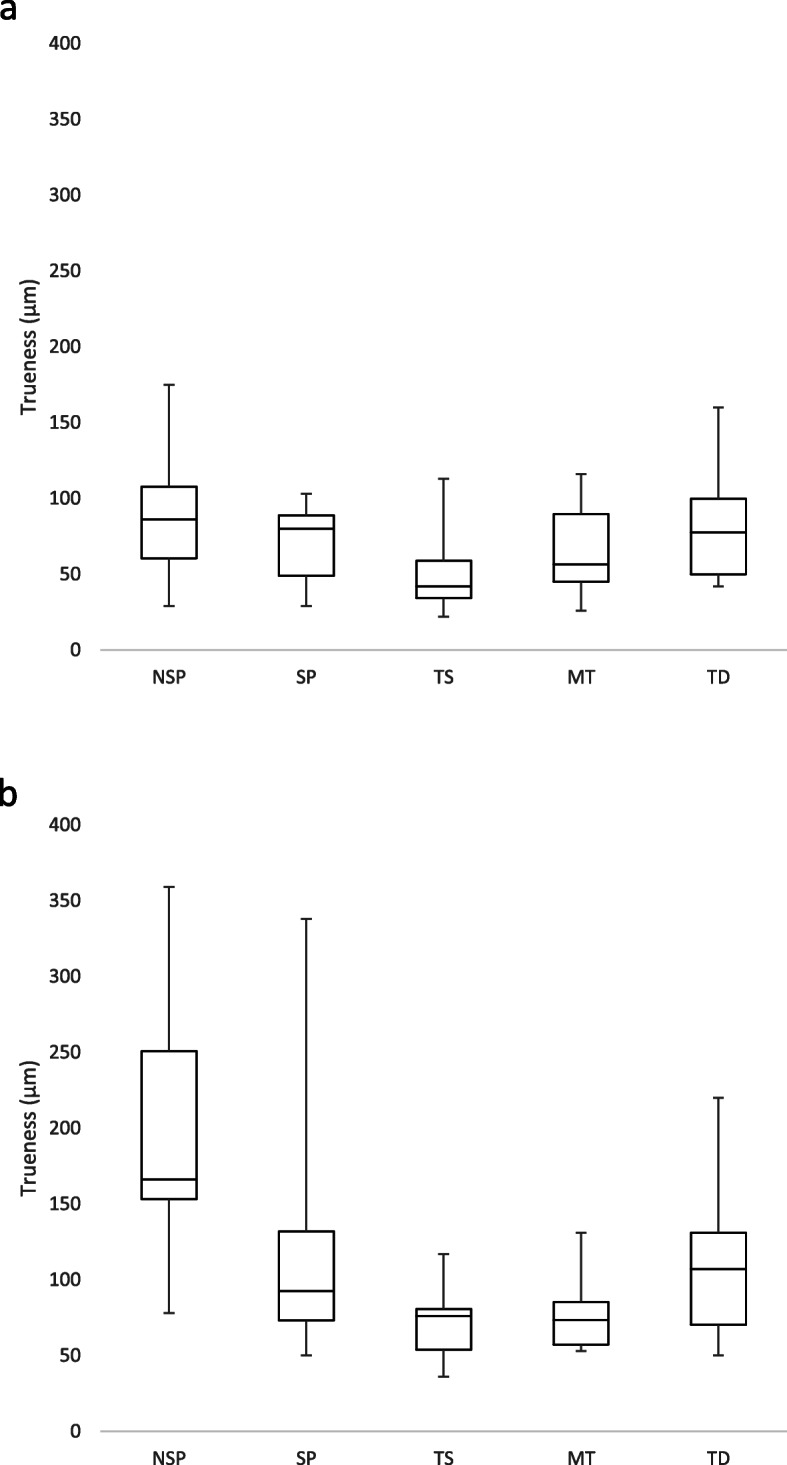
Box plot diagrams of the trueness of every impression technique. **a** Parallel implants model. **b** Divergent implants model

The results of the study were summarized in Table 2. The two-way ANOVA indicated significant interaction between impression technique and implants divergence for trueness (*p* = 0.02) and angle deviation (*p* = 0.02), but not for precision (*p* = 0.09) or inter-implant distance deviation (*p* = 0.83). In relation to trueness (Fig. 3), for the parallel implants model, the most accurate results were for TS followed by MT, TD, and SP impressions respectively. The NSP impression was least accurate. However, no significant difference was observed among the impression techniques (*p* = 0.12). For the divergent implant models, a generally similar trueness pattern was observed, with significantly increased errors for the NSP impression. There was a significant trueness difference among the groups (*p* < 0.01). However, the difference was significant between the NSP impression and all the digital impressions only (*p* < 0.05), and no significant difference was observed between the other groups. After comparing the trueness of the parallel and divergent implant models for each technique, there was a general tendency for an inferior outcome when one of the implants was divergent. However, only the NSP impression showed a significantly inferior outcome for the divergent implant models than the parallel implants model (*p* < 0.01).

**Table 2:** Summary of the trueness, precision, inter-implant distance deviation and angle deviation of every impression technique for parallel and divergent implants model

	Parallel implants model					Divergent implants model					***p*** values between parallel implants and divergent implants models				
	NSP	SP	TS	MT	TD	NSP	SP	TS	MT	TD	NSP	SP	TS	MT	TD
**Trueness**															
Mean (μm)	87.4	70.6	50.2	65.7	84.5	198.7	120.9	74.7	76.6	112.3	0.003	0.09	0.06	0.40	0.21
SD (μm)	42.4	26.8	28.2	31.2	40.3	94.2	84.5	27.2	25.2	53.7					
*p* values	All groups = 0.12					All groups < 0.001									
						NSP vs SP = 0.065		NSP vs TS = 0.001							
						NSP vs MT = 0.001		NSP vs TD = 0.031							
						SP vs TS = 0.49		SP vs MT = 0.53							
						SP vs TD = 0.99		TS vs MT = 0.99							
						TS vs TD = 0.68		MT vs TD = 0.72							
**Precision**															
Mean (μm)	105.6	119.4	56.7	99.8	119.5	169.3	137.4	84.6	129.0	131.2	<0.001	0.15	<0.001	0.09	0.48
SD (μm)	46.3	56.7	24.8	47.7	68.5	95.9	59.7	40.7	104.0	87.5					
*p* values	All groups < 0.001					All groups < 0.001									
	NSP vs SP = 0.70		NSP vs TS < 0.001			NSP vs SP = 0.34		NSP vs TS < 0.001							
	NSP vs MT = 0.98		NSP vs TD = 0.70			NSP vs MT = 0.13		NSP vs TD = 0.17							
	SP vs TS < 0.001		SP vs MT = 0.36			SP vs TS = 0.02		SP vs MT = 0.99							
	SP vs TD = 0.99		TS vs MT = 0.001			SP vs TD = 0.99		TS vs MT = 0.07							
	TS vs TD < 0.001		MT vs TD = 0.36			TS vs TD = 0.05		MT vs TD = 0.99							
**Inter-implant distance deviation**															
Mean (μm)	36.6	35.8	42.8	24.7	92.4	59.1	41.4	72.1	34.7	98.2	0.33	0.65	0.06	0.44	0.83
SD (μm)	25.0	21.9	28.2	22.4	79.4	66.3	32.7	36.0	33.5	33.1					
*p* values	All groups < 0.001					All groups = 0.01									
	NSP vs SP = 0.99		NSP vs TS = 0.99			NSP vs SP = 0.88		NSP vs TS = 0.96							
	NSP vs MT = 0.96		NSP vs TD = 0.004			NSP vs MT = 0.70		NSP vs TD = 0.25							
	SP vs TS = 0.99		SP vs MT = 0.97			SP vs TS = 0.49		SP vs MT = 0.99							
	SP vs TD = 0.003		TS vs MT = 0.84			SP vs TD = 0.03		TS vs MT = 0.30							
	TS vs TD = 0.001		MT vs TD = 0.001			TS vs TD = 0.65		MT vs TD = 0.01							
**Angle deviation**															
Mean (°)	0.5	0.3	0.3	0.3	0.3	0.8	0.4	0.3	0.4	0.4	0.003	0.23	0.95	0.07	0.14
SD (°)	0.2	0.2	0.1	0.1	0.1	0.2	0.2	0.1	0.1	0.1					
*p* values	All groups = 0.02					All groups < 0.001									
	NSP vs SP = 0.10		NSP vs TS = 0.02			NSP vs SP < 0.001		NSP vs TS < 0.001							
	NSP vs MT = 0.03		NSP vs TD = 0.03			NSP vs MT < 0.001		NSP vs TD < 0.001							
	SP vs TS = 0.53		SP vs MT = 0.73			SP vs TS = 0.04		SP vs MT = 0.76							
	SP vs TD = 0.65		TS vs MT = 0.61			SP vs TD = 0.44		TS vs MT = 0.03							
	TS vs TD = 0.77		MT vs TD = 0.83			TS vs TD = 0.08		MT vs TD = 0.60

**Fig. 4:**
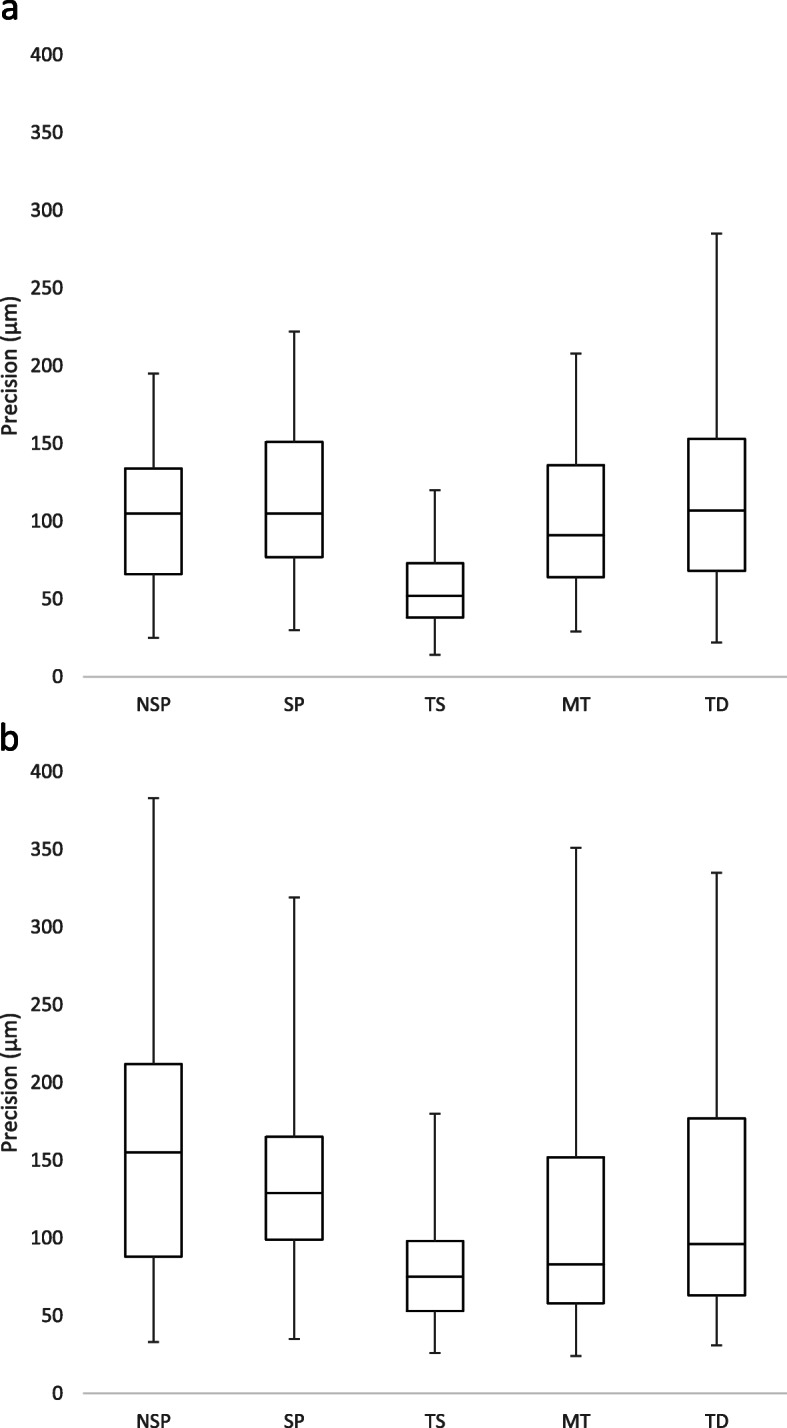
Box plot diagrams of the precision of every impression technique. **a** Parallel implants model. **b** Divergent implants model

When the implants were parallel, a significant difference in precision was observed among the impression techniques (*p* < 0.01), where only the TS was significantly more precise than the other techniques (*p* < 0.01) (Fig. 4). For the divergent implants model, significant differences in precision among the techniques were also observed (*p* < 0.01). However, this difference was between TS and NSP (*p* < 0.01); and TS and SP impressions (*p* = 0.02), but not among the different digital impressions. All the impression techniques had a more inferior precision for the divergent implants model than the parallel implants model; however, the difference was significant for the NSP (*p* < 0.01) and TS (*p* < 0.01) only.

**Fig. 5:**
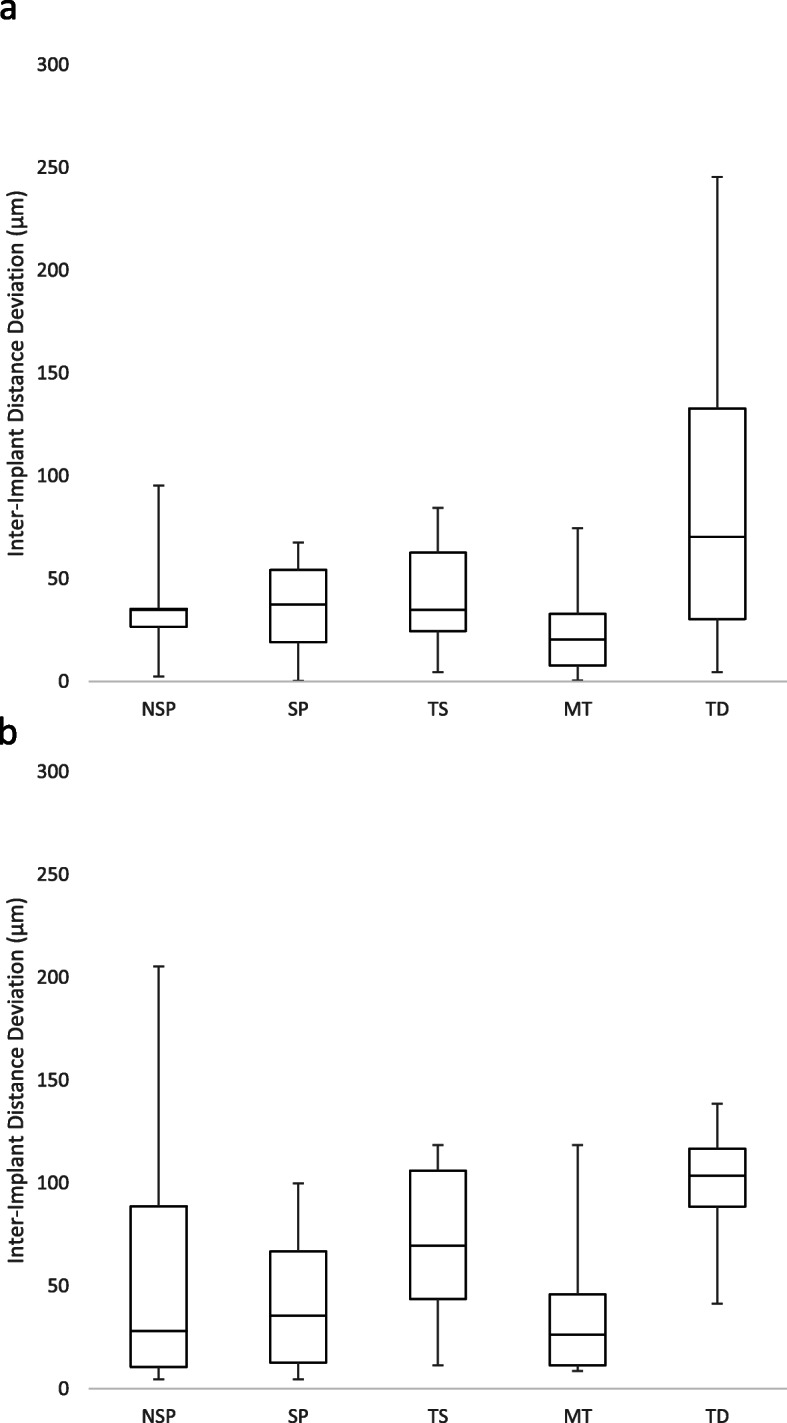
Box plot diagrams of the inter-implant deviation of every impression technique. **a** Parallel implants model. **b** Divergent implants model

Regarding the inter-implant distance deviation (Fig. 5), there was a significant difference among the groups for the parallel implants model (*p* < 0.01), where the TD had the greatest inter-implant distance deviation than all the other impression techniques, while the other techniques were generally similar. For the divergent implants model, there was a significant difference among the impression techniques (*p* = 0.01). This difference was significant between SP and TD (*p* = 0.03), and MT and TD (*p* = 0.01). For all the techniques, there was no significant difference between parallel and tilted implants in the inter-implant distance deviation.

**Fig. 6:**
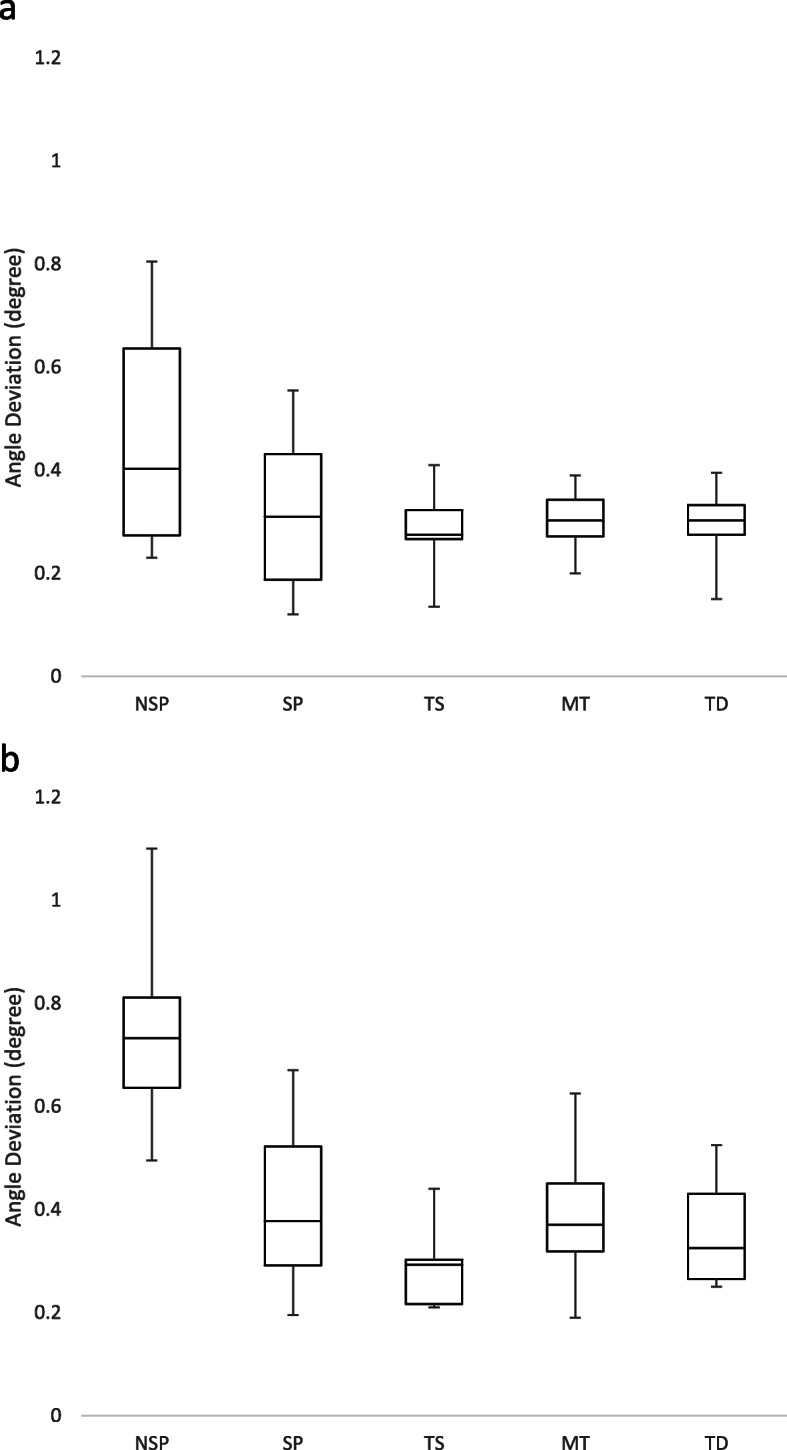
Box plot diagrams of the angle deviation of every impression technique. **a** Parallel implants model. **b** Divergent implants model

For the parallel implants model, there was a significant difference in the angle deviation among the groups (*p* = 0.02) (Fig. 6). The significant difference was between the NSP and all the digital impressions (*p* < 0.05). In general, the 2 conventional methods exhibited greater variation in angle deviation for the parallel implants model. On the other hand, the different digital impressions were generally similar. For the divergent implants model, there was a significant difference in angle deviation among the groups (*p* < 0.01). There were greater errors and variations for the NSP impression that was significantly more inferior than all other impression techniques (*p* < 0.01). TS had the least deviation, and a significant difference was found between TS and SP (*p* = 0.04), and TS and MT (*p* = 0.03). After comparing the angle deviation between the 2 models, no significant difference was observed between the impression techniques except for the NSP impressions (*p* < 0.01).

## Discussion

The results of this study indicated a general tendency for digital impressions to be more accurate than conventional impressions. This was observed for the trueness, precision, and angle deviation. This superiority became obvious for the divergent implants model, where the conventional impressions, especially the NSP technique, were more vulnerable to errors than digital impressions. Therefore, the hypotheses that there is no difference between the different digital impressions and the conventional implant impressions, and there is no effect of implant divergence were rejected. Nevertheless, the errors of all the techniques are likely to be within the acceptable clinical level (trueness of less than 200 μm). To improve the understanding of the nature of errors for the different techniques, multiple accuracy variables were included in the study [26]. A distinctive feature of the present study in comparison to earlier studies is that it converted the geometric surfaces of scanned models with the scan body to parametric surfaces that represent the implants position. This step is critical and relevant to routine application of digital dentistry. The laboratory scanning of the implant casts, and the digital impression of the implant scan body are normally conducted to generate virtual images on which the implant platform parameters are reverse engineered. Subsequently, the prosthesis framework is designed according to the reverse engineered implant platform. Therefore, evaluating the final virtual implant position is more relevant than determining solely the accuracy of scanned surface.

For the conventional impressions, the accuracy of NSP impressions was similar to SP impressions for all the evaluated variables when the 2 implants are parallel. This can be due to removing the NSP impression copings in a parallel direction to the implants which cause minimal distortion of the impression material surrounding the impression copings [3–5, 8, 17]. However, for the divergent implants model, the NSP impressions suffered from greater deterioration than SP impressions for trueness, and angle deviation. In the presence of an angle between the implants, the impression material surrounding the impression copings is deformed as the impression is removed from the model, and some of this deformation will not be fully recovered [3–5, 8, 17]. On the contrary, splinting connected the 2 impression copings together in a way that reduces the displacement of the copings within the impression during removal from the model [6]. Eventually, the impression copings were less vulnerable for individual displacement during the impression making and pouring. Further, the resin splint will prevent the rotation of the impression copings within the set impression material during fitting of the implant replicas. The superiority of the SP impression technique over the NSP impression technique has been observed by multiple earlier studies [6–8]. Nevertheless, splinting impression copings in laboratory environment may yield more accurate outcome than intraoral splinting.

In general, for parallel model, there is a similarity between the conventional impressions and the digital impressions, with the exception of angle deviation which was inferior for the NSP impressions. This supports the feasibility and merits of digital impressions for 2 implants with a 3-unit span. This corroborates the outcome of studies under in vitro conditions that confirmed similarity in outcome of digital and conventional impressions. For example, Roig et al. reported that for 2 implants with a 3-unit span, the digital impressions showed superior trueness and precision than NSP and SP conventional impressions [23]. Similarly, Papaspyridakos et al. reported a similar accuracy outcome for digital and conventional impressions for the whole arch situation [15]. For the divergent implants model, the superiority of the digital impressions became more obvious. Contrary to conventional impressions, the presence of an angle between implants seems to have a minimal effect on the accuracy of digital impressions. Most likely, this outcome is related to the digital impression not being influenced by impression material distortion during removal of impression as per the conventional impressions [16, 17]. A series of studies by Gimenez and co-workers confirmed that divergence of implants had a minimal effect on the accuracy of digital impressions [18–20]. Similarly, Lin et al., after evaluation of different implant divergence (15°, 30°, 45°), the digital impressions were associated with better accuracy for divergent implants than for conventional impressions [13]. Likewise, Papaspyridakos et al. observed that for whole arch implant scanning, implant angulation up to 15° did not affect the accuracy of the digital impressions [15]. In addition, Alikhasi et al. reported a superior accuracy of digital impressions than conventional impressions, and 45° implant angulation did not influence the accuracy of digital impressions as opposed to conventional impressions [16].

Overall, there has been a similarity between the different IOS systems used for digital impressions, which is in accordance with previous literature [21, 22]. Earlier studies reported a trueness and precision range of 10-70 μm [21, 22, 25], which is similar to the present study. The errors of the digital impressions can be attributed to scanning surface accuracy and the stitching between the different images that eventually accumulates errors with every step. The most obvious pattern of error is the inter-implant distance deviation for some of the IOS systems. This has also been reported in an earlier study where the digital impression was associated with greater inter-implant error than the conventional impression [14]. This is further accentuated by the design of the model of the study that is based on smooth ridge, which further challenged the stitching. Nevertheless, the actual magnitude of errors seems trivial (20-60 μm), and still comparable to the commonly applied conventional impression techniques. The additional source of error is the mathematical conversion of the scanned surface of the scan body to the parametric scan body and implant surfaces [12]. The minor surface irregularities may also contribute to the error in registration and subsequently the position of the implant. Eventually, this will translate in errors in final implant position. Specifically, this may be more noticeable with use of powder that may affect the surface uniformity. This may explain why in general the TD impression was the least accurate IOS system compared with the other systems. In addition, with TD being an older generation may have contributed to the inferior image outcome [21]. Several studies confirmed the superiority of newer and powderless IOS systems for short span implant scanning [21–23].

Despite that the study generally indicated a promising accuracy outcome for implant digital impression, the applied IOS systems may still have limitations that can affect its general use. This involves a lack of customization and recording of the soft tissue profile during the impression procedure [9–11]. Some reports mentioned methods to overcome this problem [9–11], which require modification of the clinical technique. It is likely that the accuracy of digital impressions in a clinical set-up is inferior to a laboratory experiment [24]. Clinically, the accuracy of digital impressions is deteriorated by the posterior location of the scan body, limited access, presence of saliva, and patient movement [24, 27]. Several studies acknowledged the effect of lack of experience on the accuracy of digital impression and the necessity of an initial learning curve to progressively ensure acceptable scanning technique [18, 19, 26, 28]. Since this study used a laboratory model, more studies are needed to validate the accuracy of IOS systems on models that resemble natural arches with teeth and variable implants positioning. A consistently reported variable that influences the accuracy of digital impression is the span of scanning, where the larger span scanning is inferior to the shorter span scanning [12, 21, 22]. Large span scanning will challenge the digital impression in obtaining an adequate image, and consistently the most terminal end tends to have the greatest error [29]. Therefore, clinical presentations of longer prosthesis span and greater number implants should be evaluated in future studies. Likewise, the effect of different implants types, connections, and scan bodies should further be investigated. Most of the studies on IOS accuracy were conducted virtually, and purely evaluating the accuracy of the generated image. Clinically, a physical cast has to be produced by milling or 3D printing to allow for prosthesis customization, veneering, and occlusal refinement. The physical cast can also be used to directly fabricate the prosthesis. Basaki et al. and Lin et al. observed an inferior accuracy of implant milled casts produced following digital impressions than conventionally produced casts [13, 14]. In addition, a significant number of casts from the digital workflow did not meet the clinically acceptable accuracy [14]. This error can be attributed to the additional processing error in fabricating the physical cast. The selected scan body of the present study, while compatible with the used implant system, was produced by a different manufacturer. This scan body type was selected because the researchers had access to the STL file of the scan body with the virtual implant, which allowed for accuracy evaluation. However, the use of components from a different manufacturer may be a source of an additional error. Therefore, the observed superior accuracy of digital impression reported in the present study should be taken with caution. While the virtual images of digital impressions seem comparable, and in certain areas, superior to the conventional, the clinical impact and actual benefits are yet to be confirmed [24].

## Conclusions

Within the limitations of the present laboratory study, it appears that the digital impressions have sufficient accuracy for the 2 implant models. There is a general tendency for the digital impressions to provide a more accurate outcome than conventional impressions, especially the NSP impression technique. The digital impressions were minimally affected by the presence of divergent angles between implants, while the NSP impression technique was most affected. Among the tested IOS systems, the TD showed the least accurate outcome.

## Data Availability

The datasets used and/or analyzed during the current study are available from the corresponding author on reasonable request.

## Abbreviations

IOS: Intraoral scanner
MT: Medit i500
NSP: Non-splinted
RMS: Root mean square
SP: Splinted
TD: True definition
TS: Trios 4
